# Draft Genome Sequences of Two Novel *Aeromonas* Species Recovered in Association with Cyanobacterial Blooms

**DOI:** 10.1128/genomeA.01181-14

**Published:** 2014-11-20

**Authors:** Mohammad J. Hossain, Roxana Beaz-Hidalgo, María J. Figueras, Mark R. Liles

**Affiliations:** a Department of Biological Sciences, Auburn University, Auburn, Alabama, USA; b Unitat de Microbiologia. Departament de Ciènces Médiques Bàsiques, Facultat de Medicina i Ciències de la Salut, IISPV, Universitat Rovira i Virgili, Reus, Spain

## Abstract

*Aeromonas aquatica* and *Aeromonas lacus* are two new species that have been found in association with cyanobacterial blooms from recreational Finnish lakes where adverse human health effects have been recorded. Here, we present the draft genome sequences of their type strains.

## GENOME ANNOUNCEMENT

The genus *Aeromonas* includes 27 species recovered mainly from aquatic environments, with several species implicated in mammalian and fish diseases (1, 2). During a survey of Finnish waters where cyanobacterial blooms were suspected to have caused adverse human health effects (i.e., fever and gastroenteritis), some *Aeromonas* strains were identified as new species, named *Aeromonas aquatica* and *Aeromonas lacus* (R. Beaz-Hidalgo, F. Latif-Eugenín, K. Berg, R. M. Niemi, J. Rapala, C. Lyra, and M. J. Figueras, unpublished data).

An Illumina MiSeq was used to generate the genome sequences for strains *A. aquatica* AE235^T^ (=CECT 8025^T^ = LMG 26712^T^) and *A. lacus* AE122^T^ (=CECT 8024^T^ = LMG 26710^T^), obtaining 1.12-Mb and 1.37-Mb sequence reads, with average coverages of 25× and 29×, respectively. The sequence reads were trimmed and assembled *de novo* using CLC Genomics Workbench (CLC bio, Cambridge, MA), generating the data shown in Table 1. The G+C content was within the range established (57 to 63 mol%) for the genus *Aeromonas* (3).

**TABLE 1:** Summary of genome data from type strains of two novel *Aeromonas* species

Strain	Accession no.	Genome size (bp)	N_50_ (kbp)	No. of contigs	G+C content (%)	No. of tRNAs	No. of proteins		
							Known function	Hypothetical	Unknown function
AE235^T^^*a*^	JRGL00000000	4,582,304	67.5	171	61.1	84	3,301	778	19
AE122^T^^*b*^	JRGM00000000	4,394,373	76.7	196	58.7	68	3,921	662	13

a *Aeromonas aquatica*.

b *Aeromonas lacus*.

The assembled genomes were annotated using the RAST server (4). The numbers of predicted tRNAs and protein-coding sequences in the two genomes are shown in Table 1. Also, the two genomes have evidence for multiple secretory systems, including type I, II, and VI, and type III was found only in AE122^T^.

The two strains are predicted to have catabolic pathways for the utilization of mannose, deoxyribose, and deoxynucleoside, d-gluconate, ketogluconates, l-rhamnose, fructose, d-galactarate, d-glucarate, d-glycerate, and d-ribose. The two genomes also encode proteins required for the utilization of glycerol, glycerol-3-phosphate, and mannitol.

The prediction of prophages using PHAST (5) demonstrated that AE235^T^ contains two complete prophages (21 kb and 77 kb) similar to *Vibrio* phages vB_VpaM_MAR and Stx2-converting phage I, respectively, and the AE122^T^ genome showed no prophages.

The draft genome sequences of these novel *Aeromonas* species are valuable additions to the genomic database that will contribute to the understanding of their role in the environment and potential pathogenicity to humans and fish.

### Nucleotide sequence accession numbers.

The draft genome sequences of the *Aeromonas* strains AE235^T^ and AE122^T^ used in this study have been deposited as whole-genome shotgun sequencing projects at DDBJ/EMBL/GenBank under the accession numbers JRGL00000000 and JRGM00000000, respectively. The version of each strain described in this paper is the first version, with accession numbers JRGL01000000 and JRGM01000000.
